# The welfare of ill and injured feedlot cattle: a review of the literature and implications for managing feedlot hospital and chronic pens

**DOI:** 10.3389/fvets.2024.1398116

**Published:** 2024-05-09

**Authors:** Emiline R. Sundman, Grant A. Dewell, Renee D. Dewell, Anna K. Johnson, Daniel U. Thomson, Suzanne T. Millman

**Affiliations:** ^1^Department of Veterinary Diagnostic and Production Animal Medicine, College of Veterinary Medicine, Iowa State University, Ames, IA, United States; ^2^Center for Food Security and Public Health, College of Veterinary Medicine, Iowa State University, Ames, IA, United States; ^3^Department of Animal Science, College of Agriculture and Life Sciences, Iowa State University, Ames, IA, United States; ^4^Department of Biomedical Sciences, College of Veterinary Medicine, Iowa State University, Ames, IA, United States

**Keywords:** animal welfare, Five Domains, feedlot cattle, production animal medicine, sickness behavior, hospital pen, chronic pen, decision-making

## Abstract

By definition, ill and injured animals are on the negative valence of animal welfare. For beef cattle kept in feedlot settings, advances in cattle health management have resulted in a greater understanding and prevention of illness and injury. However, the management of cattle once they become ill and injured is an understudied area, and there are gaps in knowledge that could inform evidence-based decision-making and strengthen welfare for this population. The aim of this review is to provide a comprehensive overview of the acquired knowledge regarding ill and injured feedlot cattle welfare, focusing on existing knowledge gaps and implications for hospital and chronic pen management and welfare assurance. Ill and injured feedlot cattle consist of acutely impaired animals with short-term health conditions that resolve with treatment and chronically impaired animals with long-term health conditions that may be difficult to treat. A literature search identified 110 articles that mentioned welfare and ill and injured feedlot cattle, but the population of interest in most of these articles was healthy cattle, not ill and injured cattle. Articles about managing ill and injured cattle in specialized hospital (*n* = 12) or chronic (*n* = 2) pens were even more sparse. Results from this literature search will be used to outline the understanding of acutely and chronically ill and injured feedlot cattle, including common dispositions and welfare considerations, behavior during convalescence, and strategies for identifying and managing ill and injured cattle. Finally, by working through specific ailments common in commercial feedlot environments, we illustrate how the Five Domains Model can be used to explore feelings and experiences and subsequent welfare state of individual ill or injured feedlot cattle. Using this approach and our knowledge of current industry practices, we identify relevant animal-based outcomes and critical research questions to strengthen knowledge in this area. A better understanding of this overlooked topic will inform future research and the development of evidence-based guidelines to help producers care for this vulnerable population.

## 1 Introduction

Animal health and welfare in food production systems is a priority for producers, retailers, and consumers (1–4). Maintaining animal welfare via promoting animal health involves many moving parts, including understanding illness and injury prevalence, minimizing illness and injury occurrence, properly treating illness and injury when it occurs, and supporting the animal during recovery. In the beef feedlot sector, ongoing research into these topics has and continues to make improvements in feedlot cattle health and welfare. Prevalence studies are essential for understanding how common a particular condition is and how it is distributed across populations of interest. Prevalence studies have been conducted on feedlot conditions such as digital dermatitis (5), lameness (6), *Mycoplasma Bovis* (an important respiratory pathogen for Bovine Respiratory Disease Complex [BRDC]); (7), and ruminal acidosis (8). These studies have important implications for planning health management and research needs. Vaccination is one of the most crucial tools for preventing feedlot cattle infectious disease (9). Effective vaccination programs to lessen disease occurrence include considerations for vaccine type, vaccination timing, and secondary risk factor management (10–14). Understanding risk factors associated with illness and injury is also vitally important. For example, BRDC is a complex disease with risk factors that include host immunity levels, environmental conditions, and bacterial and viral pathogens that can influence pathogen transmission and stress-induced susceptibility (15). Risk factors associated with feedlot cattle lameness include body weight, source, stocking density, percentage of forage in the diet, season, precipitation, and temperature (16). By knowing and understanding how these risk factors impact feedlot cattle illness and injury occurrence, producers can work to minimize their herd’s exposure to these risk factors, thus decreasing their risk of illness or injury.

In contrast, managing individuals after illness or injury has occurred is a less studied topic that has considerable implications for cattle welfare. Feedlot audits such as those in the United States (U.S.) (17), Canada (18), and under development in Australia (19) include sections addressing ill and injured cattle populations housed in hospital or chronic pens, and the U.S. and Canada audits indicate that failure to euthanize critically ill/distressed or injured cattle in a timely manner is an egregious act of neglect that can result in audit failure. Additionally, the care of ill and injured cattle has important implications for beef production sustainability and future. The Global Roundtable for Sustainable Beef^1^ has identified animal health and welfare among core principles necessary for a viable beef value chain. Ill and injured cattle management and decision-making has implications for the economic viability of feedlot operations, social license to farm, and sustainability of the feedlot and beef sectors over time. Thus, there is considerable synergy between enhancing ill and injured feedlot cattle welfare and supporting feedlot operation productivity and sustainability. However, despite the growing importance of ill and injured feedlot cattle management and welfare, evidence-based guidelines designed to strengthen the care and welfare of ill and injured cattle are lacking. Thus, the aim of this review is to provide a comprehensive overview of the acquired knowledge regarding ill and injured feedlot cattle welfare, with a focus on gaps of knowledge that exist and implications for hospital and chronic pen management and welfare assurance. To achieve this, we will outline the current understanding of the cattle that make up this population. Then, we will appraise the impacts of illness and injury on cattle welfare using the Five Domains Model (20) and suggest how this model can guide future research. Ultimately, a better understanding of this overlooked topic will inform evidence-based guidelines for best practices in managing ill and injured feedlot cattle to help producers support the welfare of this vulnerable population.

## 2 Literature review methodology

A literature review was completed to understand the current published scientific findings specific to welfare and management of ill and injured feedlot cattle. Three separate searches were conducted. The first search was designed to identify peer-reviewed papers reporting on the welfare of ill and feedlot cattle. The second search focused on identifying peer-reviewed papers reporting on managing cattle in feedlot chronic pens. The third search was then widened to include feedlot hospital pens and other specialty pens used to house ill and injured cattle. After the initial search, papers deemed to be irrelevant were removed using the following exclusion criteria: “dairy” in the topic (Web of Science) or Article title, Abstract, or Keywords (Scopus) of the paper, “other topic” (about feedlot cattle, but no illness or injury animal outcomes), or “other reasons” (species other than cattle, non-peer-reviewed sources, language other than English). Detailed methodology, such as the specific search terms and databases used, the number of papers excluded for each exclusion criteria, and the final results from these searches (total and by paper type) can be seen in Table 1.

**Table 1:** Results from three literature searches on the welfare and management of ill and injured feedlot cattle.

	Raw Results (#)	Excluded for “dairy” topic^1^ (#)	Excluded for “topic – other”^2^ (#)	Excluded for other reasons^3^ (#)	Total excluded (#)	# remaining by article type (including repeats)			# remaining by article type (excluding repeats)			Total relevant papers (#)
						Primary Research	Review	Other^4^	Primary Research	Review	Other^4^	
Search 1: (“cattle” OR “beef cattle” OR “calf” OR “calves”) AND (feedlot OR “feed lot” OR feedyard OR “feed yard” OR “dairy”) AND (welfare) AND (sick OR sickness OR ill OR illness OR injured OR impaired OR unhealthy OR invalid OR ailing OR diseased OR down OR downer OR downed OR wounded OR damaged OR disabled OR lame OR emaciated OR debilitated)												
Web of Science (All databases, topic search)	4,787	4,596	65	23	4,684	77	14	12	82	14	13	110
Scopus (Article title, abstract, keywords search)	553	529	8	0	537	13	3	1				
Search 2: (“cattle” OR “beef cattle” OR “calf” OR “calves”) AND (feedlot OR “feed lot” OR feedyard OR “feed yard” OR “dairy”) AND (“chronic pen” OR “chronic pens”)												
Web of Science (All databases, topic search)	2	0	0	0	0	1	0	1	1	0	1	2
Scopus (Article title, abstract, keywords search)	2	1	0	0	1	1	0	0				
Search 3: (“cattle” OR “beef cattle” OR “calf” OR “calves”) AND (feedlot OR “feed lot” OR feedyard OR “feed yard” OR “dairy”) AND (“hospital pen*” OR “sick pen*” OR “specialty pen*” OR “railer pen*” OR “realizer pen*” OR “recovery pen*” OR “special needs pen*” OR “alternate pen*” OR “alternative pen*”)												
Web of Science (All databases, topic search)	59	46	1	1	48	11	0	1	11	0	1	12
Scopus (Article title, abstract, keywords search)	35	25	0	0	25	10	0	0				

1 Papers with “dairy” in the topic were removed using the “NOT” Web of Science advanced search option. Papers with “dairy” in the Article title, Abstract, or Keywords were removed using the “AND NOT” Scopus advanced search option.

2 “Topic – other” was defined as papers about feedlot cattle that had no ill or injured animal outcomes.

3 “Other reasons” was defined as papers about non-cattle species, with non-peer-reviewed sources, or in non-English languages.

4 The “other” article type included conference proceedings, meeting abstracts, book chapters, or opinion/editorial material.

In summary, 110 unique articles about the welfare of ill and injured feedlot cattle were identified. The inclusion criteria for this search were quite broad—any papers that mentioned welfare, feedlot cattle, and measured any illness or injury animal outcome or discussed applications for ill or injured cattle. Applying more specific criteria such as restricting the scope to studies explicitly conducted on ill or injured cattle would likely further decrease this number. The literature search also identified 12 unique articles about managing ill and injured cattle in specialized hospital-type pens and two about managing them in chronic pens. Due to the sparsity of papers identified in this literature search, this review was further supplemented by papers identified through other manual methods. These manual methods included searching through reference lists from the original results, performing less targeted literature searches, searching journals associated with feedlot production or medicine, and talking to North American feedlot cattle experts for paper recommendations. Due to the limited number of papers on ill and injured feedlot cattle, some published papers exploring these topics in dairy cattle that had been excluded from the literature results table are included within the discussion to provide a more holistic view of the state of the literature on ill and injured cattle management.

It is important to acknowledge potential sources of bias in our methodology. Limiting the literature search publications in the English language may have biased the results towards articles from English-speaking countries. Inclusion of the search terms associated with housing (i.e., feedlot and feedyard) likely excluded results from extensive housing systems and pasture-based systems. Additionally, while articles from outside of North America were identified during the literature search and included, the manual search methods may have been biased towards North American intensive feedlot systems.

## 3 Defining ill and injured feedlot cattle

### 3.1 Ill vs. injured cattle

According to the Merriam-Webster dictionary, the definition of impaired is “in an imperfect or weakened state or condition” (21). In this review, impaired cattle will be defined as those in weakened states or conditions compared to healthy, fully functioning cattle, regardless of the source (injury, disease, other) or severity (mild, severe, acute, chronic). The definition of “impaired” includes two main subcategories—“ill” (synonyms: sick, unwell) (22) and “injured” (synonyms: damaged, wounded) (23). Thus, ill cattle are considered as those not in good health due to disease or other pathological conditions and injured cattle as those with physical harm or damage to the body not attributed to disease.

According to the United States Department of Agriculture (USDA) National Animal Health Monitoring System’s (NAHMS) Feedlot 2011 survey of the U.S. feedlot industry, the most common conditions in feedlot cattle in operations ≥1,000 head are: respiratory disease (16.2% of cattle), digestive problems (4.3% of cattle), acute interstitial pneumonia (2.8% of cattle), bullers (2.8% of cattle), lameness (1.8% of cattle), and central nervous system problems (1.1% of cattle; e.g. polio) (24).

### 3.2 Acute vs. chronic cattle

For this review, “acute” and “chronic” cattle are considered two separate subcategories of impaired cattle. Acute cattle are those with conditions that resolve within a short time (days or weeks), either successfully through recovery or unsuccessfully through mortality. Acute cattle are often treated in their home pen or may be temporarily housed in a treatment/hospital pen before being returned to their home pen. Conversely, chronic cattle are those with long-term conditions (weeks or months) that result from failure to recover in a timely manner. Chronic cattle are often treated multiple times and may be moved to a separate chronic pen after failed treatments.

Data regarding acute vs. chronic cattle prevalence is lacking in the published literature, and most data that is available pertains to respiratory disease. The USDA NAHMS 2011 U.S. feedlot survey data for feedlots ≥1,000 head (24) reported expected percentages of cattle for each final disposition (recovery, mortality, chronicity, and retreatment) after one, two, or three treatments for respiratory disease in two different weight classes (above or below 318kgs [700lbs] when placed). Regardless of weight class, approximately 16.2% of feedlot cattle were diagnosed with respiratory disease, and 87.5% of those were treated. Of those treated, greater than 80% of cattle recovered after one treatment (and hence were categorized as acute cattle according to our definition), and mortality rate after first treatment was less than 4%. Of the treated cattle, less than 15% received additional treatments, and additional treatments were often with a different product. Successful second treatment response was lower than the first treatment response (over 60%), and mortality for second treatment cattle was also higher (about 13%). Finally, a small percentage of cattle fail to respond to both the first treatment and second treatment. At this stage, producers may decide to pursue further treatment or other alternatives, such as railing (shipping for slaughter prior to reaching expected slaughter weight) after an appropriate antibiotic withdrawal period. The third treatment response rate (~40%) was lower than both the first and second treatment response rates, and the mortality rate (~30%) for third treatment cattle was also higher. This higher mortality rate can be expected and is perhaps due to factors such as infections with drug-resistant pathogens or because the disease has progressed to a severe point where the animal cannot adequately respond to the infection or recover their respiratory function (24). Additional treatments beyond the third treatment are not reported in the NAHMS data, but casual observation indicates that this population does exist at some feedlots.

**Figure 1:**
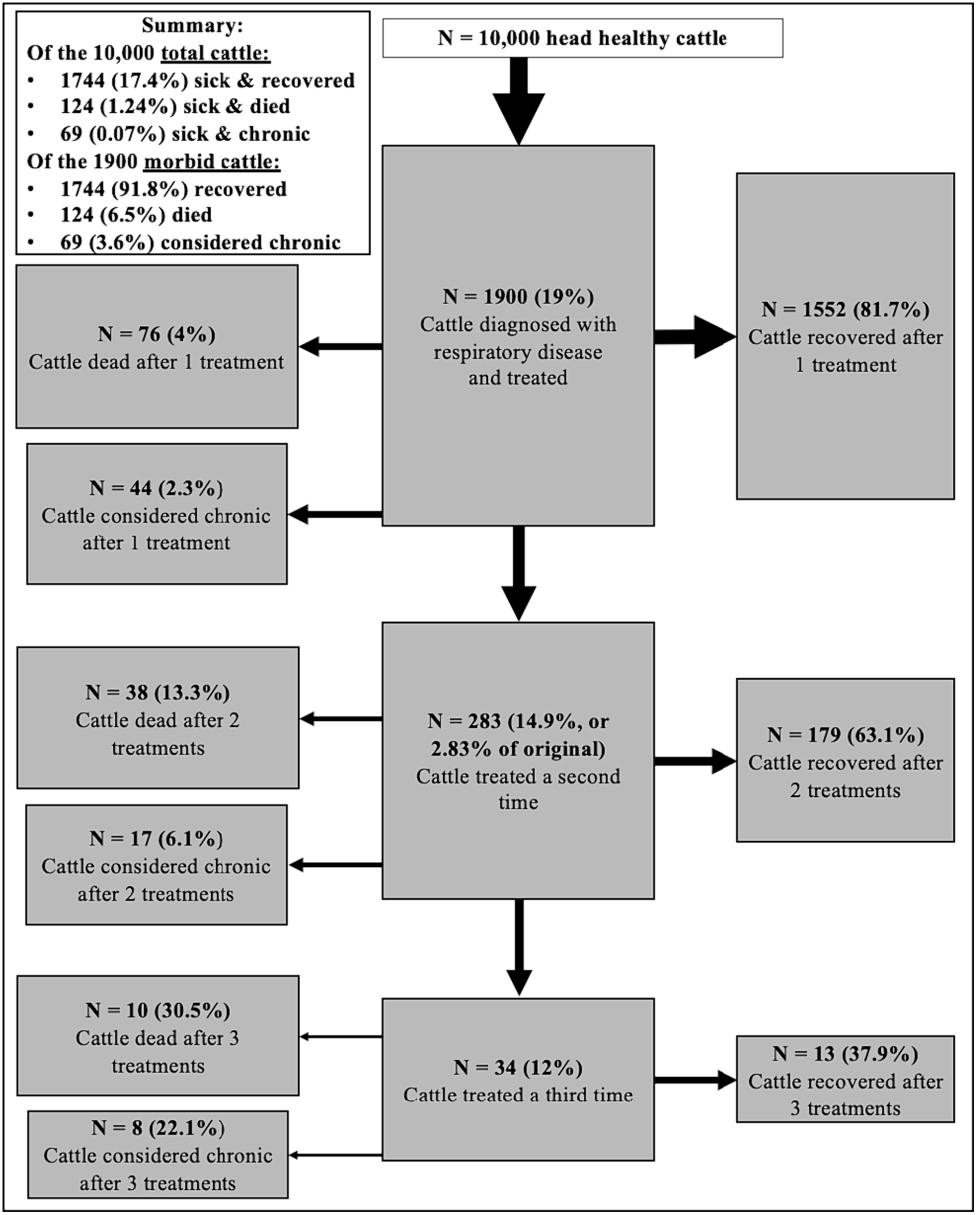
Dispositions for number (N) and percent (%)^1^ of feedlot cattle diagnosed with respiratory disease after one, two, and three treatment events in a hypothetical feedlot of 10,000 head. All cattle were < 318kgs (700lbs) when placed. Adapted from USDA NAHMS^2^ data (24) for US feedlots ≥1,000 head. ^1^Percents may not add to 100 due to multiple or unspecified responses. ^2^United States Department of Agriculture (USDA) National Animal Health Monitoring System (NAHMS) Feedlot 2011. ^3^Considered chronic and realized (railed). Defined as cattle shipped for slaughter before reaching expected slaughter weight.

Figure 1 presents these data specific to a hypothetical feedlot of 10,000-head of cattle <318kgs (700lbs) when placed, which have a higher respiratory disease morbidity (21.2%) and treatment (19%) rate than cattle ≥318kgs (700lbs) when placed (8.8% morbidity and 7.4% treatment rate). In summary, a 10,000 head feedlot with cattle placed at <318kgs (700lbs) will treat approximately 1900 cattle for respiratory disease. Of those 1900 cattle treated, 1744 (91.8%) will recover, 124 (6.5%) will die, and 69 (3.6%) will be considered chronic and railed within one, two, or three treatment events (these numbers are slightly above 100% of cattle, due to multiple responses in the NAHMS data). Relating these numbers to the original population of healthy cattle, 17.4% will be diagnosed with respiratory disease, treated, and recover. The expected mortality rate for respiratory disease would be 1.24%, with only 0.07% of cattle being diagnosed with respiratory disease, treated, and becoming chronic. The “total outs” (mortality + chronicity) from respiratory disease would be 1.93% of the original population. While this figure outlines cattle outcomes for respiratory diseases, some questions remain. For example, what happens to cattle that fall under each of these outcome categories, and how severely and how long is their welfare impacted? What are the implications for impaired cattle with conditions besides respiratory disease?

### 3.3 Dispositions and welfare considerations

There are four common dispositions that both acutely and chronically ill cattle may experience: recovery, railing, euthanasia, and unassisted death. For both the producer and the animal, the best-case outcome is recovery. Estimates for mortality rates in feedlots range from 1 to 2% (24–26), meaning that the vast majority of morbid cattle will recover (with recovery defined as not a mortality event). Precise numbers of ill or injured cattle that will fully recover are sparse. The NAHMS data indicates that of the 16.2% of cattle affected by respiratory disease, approximately 92–96% will recover after 1–3 treatment events (92% of cattle <318 kgs [700lbs] when placed and 96% of cattle ≥318 kgs [700lbs] when placed, respectively). The data also suggests that chronic cattle with BRDC (which often receive multiple treatments) may have decreased recovery rates. A descriptive epidemiologic report of chronic calves from a single Western Canadian feedlot in 1998 reported that 60% of calves in their chronic pen were returned to their home pen after an average recovery period of 30 days (27). More recently, a small study completed in Iowa feedlots found that 83% of the calves that entered the chronic pen were either returned to their home pen or harvested from the chronic pen (28). While variable geographic and climactic conditions may explain some of these differences, the overall sparse and variable reports of cattle recovery rates in different feedlots suggests that animal recovery is also likely impacted by factors within the feedlots themselves, and more research is needed into what factors can impact animal recovery.

Railer cattle (sometimes called “realizer” cattle) are cattle sold before reaching their expected slaughter weight. Reasons for this failure to reach slaughter weight can include injury, chronic illness, poor performance, or a combination of these factors. The term “railer” stems from the ultimate endpoint– the rail at the packing plant. Cattle sent to slaughter as railers are expected to have a lighter-than-typical carcass weight, which leads to some losses on the initial investment (29). Very little data on the expected number of railer cattle in a feedlot setting is available. One source indicates that the expected proportion of cattle in a feedlot that will be railed is 0.42% (30). According to Terrell and colleagues, the leading diagnosis of railer animals is lameness and skeletal issues (47.83% of railers), followed by BRDC (43.48% of railers) and non-performance issues (8.7% of railers) (31). There is an economic incentive for producers to rail animals for partial value, but animal welfare considerations must also be considered. Railer cattle that are ill and or/injured may be in a negative state of animal welfare due to their illness or injured state and may be experiencing negative mental states such as pain, distress, or feelings of malaise. The additional stressors involved with the railing process (such as transport, being sold at auction, and adapting to a new environment) may be exacerbated in ill and injured cattle compared to healthy cattle. Cattle should only be railer candidates if they are not in pain, can freely stand and walk, and are disease, drug, and chemical residue free (32). Cattle should also meet fitness for transport guidelines, such as those outlined by the American Association of Bovine Practitioners (AABP) and Canadian Food Inspection Agency fitness for transport guidelines (33, 34). Thus, when deciding whether to keep an ill or injured animal with the hope of recovery, market it as a railer animal, or potentially euthanize it if it is not fit for transport, there are welfare risks that need to be considered (35, 36).

Some animals may be so severely ill or injured that euthanasia is the best option. Guidelines for humane euthanasia of cattle are provided by AABP, Beef Quality Assurance, and the American Veterinary Medical Association in the U.S. and by the National Farm Animal Care Council in Canada. When deciding which animals are candidates for euthanasia, factors to consider include the animal’s pain and distress, quality of life, likelihood of recovery, ability to get to feed and water, drug withdrawal time, economic considerations, condemnation potential, human safety, and diagnostic information (37–41). The AABP guidelines suggest that no more than 4 h should pass between the euthanasia decision and the euthanasia event (41). Euthanasia decisions are a key evaluation criterion in feedlot audits, including those created by the National Cattlemen’s Beef Association in the U.S. and the National Cattle Feeders Association in Canada. Failure to euthanize a distressed animal in a timely manner is considered an egregious act of neglect, which results in an automatic audit failure (17, 18). This possibility of an audit failure from the identification of an animal in need of euthanasia that is not being properly handled highlights the importance of timely euthanasia and clear protocols to provide guidance about when and how to euthanize a severely ill or injured animal. While timely euthanasia is a crucial factor that can impact animal welfare, there is evidence that timely euthanasia may not always occur at necessary levels. Timely euthanasia is subjective, and data is limited; more guidance exists for dairy cattle than feedlot cattle. A 2020 review on timely euthanasia in the dairy industry concluded that timely euthanasia is a concern in the dairy industry and that more resources are needed to provide employees with the tools necessary to make these critical decisions (38). For example, dairy caretakers that underwent case-study based training for treatment decisions such as euthanasia stated that while they felt confident in making euthanasia decisions before the case study discussion, the training experience was still beneficial in improving their euthanasia decision-making skills (42). A 2019 survey of pen riders from 31 Texas feedlots indicated that feedlot pen riders are less confident in performing euthanasia than managers and veterinarians and were more likely to indicate that cattle are not always euthanized in a timely manner (43). Primary research focusing on clarifying ambiguity in euthanasia guidelines and increasing confidence in euthanasia decision-making by providing clear animal-based outcomes and defined endpoints are needed to ensure timely euthanasia of ill and injured feedlot cattle. Additionally, the practice of on-farm emergency slaughter (OFES) is used as an alternative to euthanasia in some countries for cattle that are unfit for transport but still fit for human consumption (44). While OFES is intended to prevent transportation of unfit animals while salvaging their meat (45), there is some controversy over whether OFES provides prompt relief (i.e., quickens or delays death) for injured animals (46, 47), and thus additional research in necessary to determine the welfare implications for cattle undergoing this process.

Unassisted death occurs when an animal dies without human intervention (i.e., in the absence of euthanasia). While unassisted deaths are more common than assisted deaths (euthanasia) in U.S. feedlots, overall mortality rates are low at only 1–2%. Hence, unassisted death is a relatively uncommon outcome overall for feedlot cattle. Unassisted death may result from acute conditions (e.g., heart failure, lightning strikes) or chronic conditions (i.e., chronic BRDC or lameness). A key welfare consideration for cattle that die unassisted is the severity and duration of suffering before a mortality event. This is especially true for chronically ill or injured cattle, since chronic impairments that have progressed to a state of severity where death is imminent are likely accompanied by severe welfare impairments (such as pain, distress, breathlessness, malaise, hunger, and discomfort). Minimal information on the prevalence of unassisted deaths in chronic cattle is available. One reason for this may be that some feedlots or sources may not indicate whether mortalities result from euthanasia vs. unassisted death. For example, Pollock and colleagues indicate that for chronic calves, 40% either died or were euthanized after a short recovery period of only 15 days (27), but does not further split this into unassisted deaths vs. euthanasia. The limited information available indicates that unassisted death is typically more common than euthanasia. A small study of 5 Iowa feedlots reported 14% mortality, and 3% were identified as euthanized. When asked, most feedlot managers responded that unassisted deaths were more frequent in their chronic pens than assisted deaths (euthanasia) (28). Research regarding factors that may lead to non-responsive cases, unassisted death, and animal-based outcomes that are indicators for immediate euthanasia is an area of need that could help clarify euthanasia guidelines, barriers to timely euthanasia and ultimately minimize the occurrence of unassisted deaths.

### 3.4 Behavior during convalescence

To understand why ill and injured cattle may benefit from specialized care and management, it is first necessary to outline how behavior differs between impaired and healthy animals. It is well established in the scientific literature and clinical practice that when an animal is ill or injured, its behavior will change. In the past, sickness behaviors were considered an undesirable disease effect. In a critical review in 1988, Hart described changes in animal behavior as a response to sickness not as a “maladaptive or undesirable effect of illness, but rather a highly organized behavioral strategy that is at times critical to the survival of an individual” (48). In other words, the function of sickness behavior is integrated with the innate immune response, which influences an animal’s chances of recovery from illness. Research regarding sickness behavior as an adaptive response to disease in humans and other animal species has continued to grow [for more recent reviews, see (49–52)].

Activating the innate immune system is the first step of many that ultimately leads to a change in impaired animal behavior. Neuroimmunoendocrine mechanisms behind sickness behavior have been an active area of study [e.g., (49, 50)]. To summarize, the innate immune system can be activated in response to infection with a pathogen, tissue damage, and other irritants (e.g., heat stress) (52). When an animal is infected with a pathogen, immune cells recognize molecular structures on the pathogen called pathogen-associated molecular patterns (PAMPs). When an animal experiences tissue damage, the broken cells produce alarmins. In both of these cases, sentinel immune cells such as dendritic cells, macrophages, and mast cells have receptors that can detect PAMPs or alarmins and will respond with the release of inflammatory cytokines, which are the primary agents that result in what we call sickness behaviors (50, 52). Four major cytokines are associated with sickness behavior: Tumor necrosis factor-α (TNF-α), Interleukin-1 (IL-1), Interleukin-6 (IL-6), and high mobility group box protein-1 (HMGB-1) (50, 53, 54). There is some evidence that other cytokines, such as interleukin-18 (IL-18) and interferon-γ (IFN-γ), may also play a role in sickness behaviors (50, 55). These inflammatory cytokines act on the brain to trigger responses that include physiological changes in the body (such as fever) and sickness behaviors.

Many behavioral changes occur that are considered sickness-related behaviors, and these behaviors are highly conserved across animal species. For a recent review on non-species-specific sickness behaviors, see (56). Most primary research on cattle sickness behavior has been studied in dairy cows with common dairy production diseases, such as hypocalcemia, ketosis, metritis, mastitis, and lameness. Sick dairy cows displayed increased resting/lying duration (57–59), decreased activity (60, 61), and decreased feeding behaviors [e.g., time at feeder, number of feeder visits, feed intake (59, 62–65)]. Sick dairy cows also expressed decreased duration ruminating (60, 61). Social behavior expression also decreased in response to sickness. For example, sick dairy cows performed fewer bunk displacements (64), fewer agonistic behaviors (65, 66), and less allogrooming (66). One study also reported that lame dairy cows were recipients of social licking by their pen-mates more frequently than non-lame dairy cows (67). Neonatal dairy heifer calves infected with Bovine Respiratory Disease Complex (BRDC) and neonatal calf diarrhea displayed decreased exploratory behavior when exposed to novel object and stationary human approach tests relative to healthy calves (68). When exposed to a low dose of bacterial endotoxin, dairy calves expressed sickness behaviors such as decreased rumination, decreased hay eating, decreased self-grooming, increased lying, and increased standing inactive (69). For a more extensive review of dairy cattle sickness behaviors, see (53). These sickness behaviors in dairy cattle may have some application for beef cattle. Nevertheless, research on sickness behaviors in beef feedlot cattle specifically, including male cattle, is necessary due to differences in genetics, nutrition, environment, and rearing that may impact these behaviors.

Primary research on feedlot cattle sickness behaviors is less extensive and primarily focused on BRDC. Cattle with BRDC display decreased activity (70–72), decreased feeding behaviors [e.g., lower dry matter intake, less time feeding, and less time near the bunk; (73–76), decreased rumination (70, 72), fewer lying bouts (71, 73), and increased lying duration (73)]. Cattle with BRDC may also groom less (73) and may have a lower pain threshold [hyperalgesia; (73)]. These general sickness behaviors may also be expressed in cattle with other common feedlot diseases, such as acidosis, pneumonia, digital dermatitis, and general lameness. For example, a review article on feedlot cattle with acidosis states that decreased feed intake is a consistent clinical sign of cattle with acidosis (77). Cattle with pneumonia spent more time lying down and less time eating than healthy counterparts (78). Cattle with digital dermatitis showed decreased rumination and increased inactivity (79). Like dairy cattle, there is evidence that diseased feedlot steers may receive more allogrooming than their non-diseased counterparts (80). There is also some evidence that water intake will change with disease and can be used to predict disease onset (81). Finally, level of parasitic infection (severity of disease) can impact the level of sickness behavior expressed by an animal (82–84).

### 3.5 Identifying and managing ill and injured feedlot cattle

Ill or injured feedlot cattle are identified by employees called pen riders. At a larger feedlot, pen riders are typically a separate group of employees responsible for checking pens and identifying ill or injured animals, sometimes from horseback (hence the term “rider”). At a smaller feedlot, while there may not be a designated “pen rider” job, there are still employees responsible for regularly checking cattle pens. Pen riding is a difficult task, and it requires excellent observation skills and knowledge of what to look for to identify individual ill or injured cattle in large groups of healthy cattle. Portillo provided a comprehensive description of the best practices in pen riding in U.S. feedlots, including how season, cattle excitability, and cattle risk status can impact pen riding strategies (85). Recent technological advances have also made identifying ill or injured cattle with technology feasible, although this is still a developing area (86). Once an animal is identified as ill or injured and in need of treatment, treatment strategies for that animal may vary depending on the disease identified, the etiology and severity of the disease, and the characteristics of the affected animal.

It is important to recognize that ill and injured animals are unavoidable in livestock production. While the ideal situation would be that all animals remain healthy, and producers, veterinarians, and researchers continue to strive for this goal, ill and injured cattle exist. Thus, when ill and injured cattle are identified, it is vital to manage them in a way that promotes positive and minimizes negative welfare while supporting their return to health. An ill or injured animal is inherently on the negative valence of animal welfare (experiencing a negative rather than positive state). Careful and thoughtful management practices can promote and support cattle welfare while they are impaired. The practices and types of pens used for housing and managing ill and injured cattle populations vary greatly between feedlots. For this review, any pen specifically designated to house impaired cattle of any kind will be defined as a pen in a “hospital pen system.” Within a hospital pen system, there are three sub-categories of pen type: “hospital pen,” “chronic pen,” and “specialty pen.” A hospital pen houses acute cattle for a short stay, and cattle have often been recently treated. The chronic pen typically houses chronic cattle for a longer stay compared to the hospital pen. Cattle in this pen often have been treated multiple times and may or may not receive additional treatments (87). Finally, a specialty pen is any pen that does not fit within the hospital or chronic pen designations. Examples of specialty pens in feedlots include the buller pen (which houses cattle affected by buller-steer syndrome) and the realizer or railer pen (which houses cattle that will be shipped to slaughter before reaching market weight). Some facilities may have separate or combined chronic and railer pens, and others may have an additional extended-recovery pen or small pasture for animals that would typically be housed in a chronic/railer pen but may benefit from additional time in a recovery pen instead of being immediately railed once drug withdrawals are met. The number and types of pens in a feedlot hospital pen system will vary depending on the feedlot size and needs. Feedlot purchasing practices, such as a predominance of higher or lower-risk cattle, may also influence hospital pen systems. A large feedlot or one with a large high-risk cattle demographic may have enough morbid animals to support many pens in their hospital pen system for different types of impaired cattle. In contrast, a smaller feedlot or one that purchases lower-risk cattle may only have a single pen for all impaired cattle (87, 88).

## 4 The Five Domains and ill and injured cattle welfare

### 4.1 The Five Domains Model—an introduction

**Figure 2:**
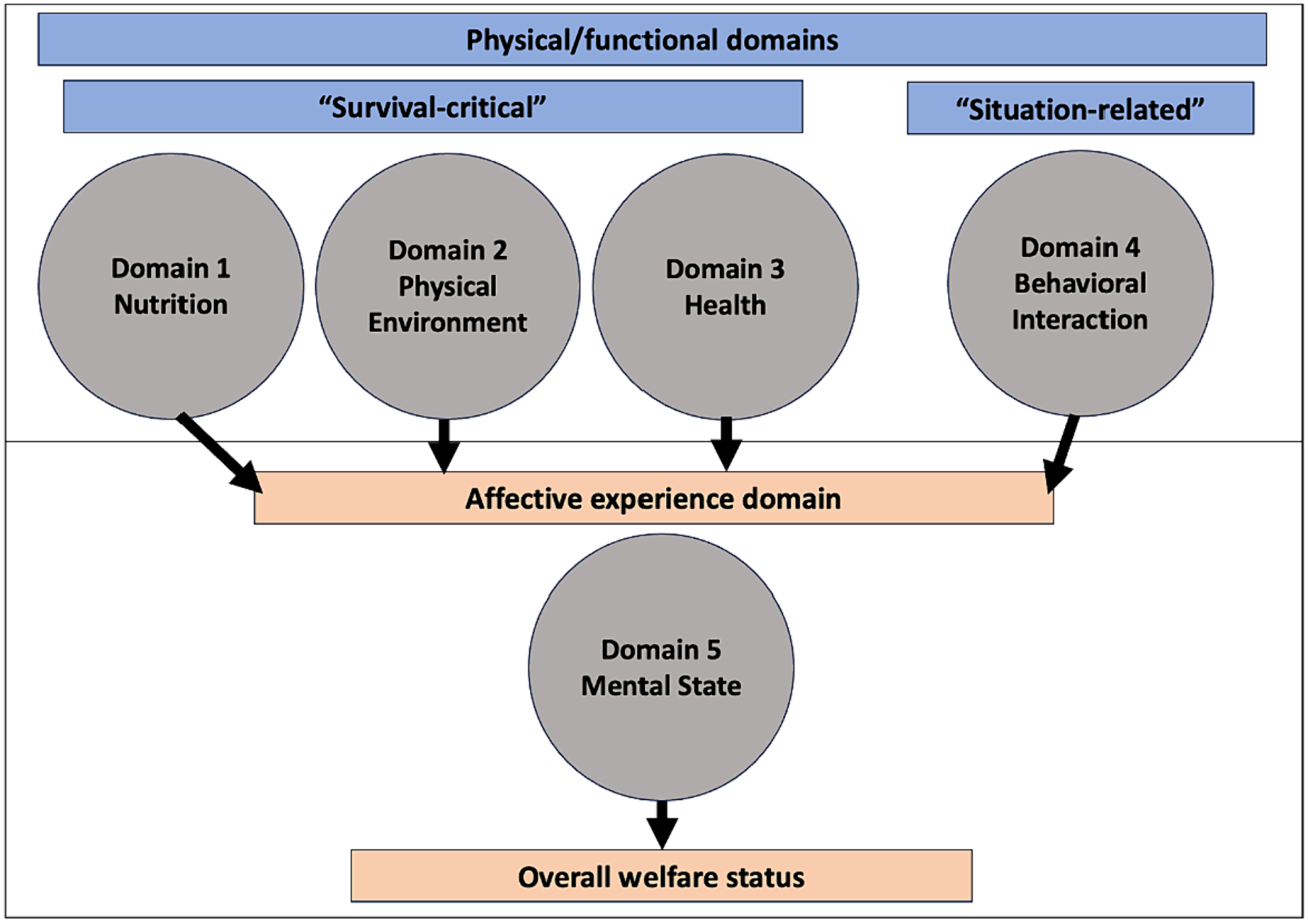
A diagram of the Five Domains Model of animal welfare. Adapted from Mellor et al. (20); licensed under CC BY 4.0.

According to the Terrestrial Animal Health Code published by the World Organization for Animal Health (WOAH), animal welfare is defined as “the physical and mental state of an animal in relation to the conditions in which it lives and dies” (89). The Five Domains Model is a conceptual framework and tool for assessing animal welfare. Introduced by Mellor and Reid in 1994 (90), the Five Domains Model contains five areas (Domains): 1- Nutrition, 2- Physical Environment, 3- Health, 4- Behavioral Interaction, and 5- Mental State (Figure 2). The first four domains are considered physical/functional domains, as they focus on the internal physical state of the animal. Domain 5 is the mental state domain, as it considers the mental experience of the animal and how the aspects of the first four domains impact that animal’s mental state. Therefore, the first four domains are filtered through the mental state domain to ask, “how do these functional domains impact the animal’s subjective mental experience?” or more simply, “how do they make the animal feel?.” The overall mental state of the animal as a cumulation of the impacts of the first four domains can then be used to assess the animal’s current welfare state (20).

The first three domains (Nutrition, Physical Environment, and Health) are often referred to as the “survival-critical” domains, as they give rise to negative affect (Mental State Domain) critical to the animal’s survival (such as breathlessness, thirst, hunger, pain, nausea, dizziness, and weakness) (91). Domain 4 (Behavioral Interaction) focuses on an animal’s external physical and social environment and how behavioral interactions with the environment can impact welfare. These situation-based factors considered in Domain 4 reflect the cognitive responses of animals in different situations, such as being kept in impoverished environments, confronted by threatening situations, or otherwise restricted in their ability to engage in agency-related behaviors (20, 91). Agency is defined as an animal’s ability to consciously engage in goal-directed behaviors, or more simply its ability to choose the behaviors it expresses (20). Situations where agency is impeded may cause negative affect (Mental State Domain) such as anxiety, fear, panic, frustration, anger, helplessness, loneliness, boredom, and depression. Situations where agency can be exercised may cause positive affect (Mental State Domain) such as calmness, engagement, excitation/playfulness, and confidence (91). There are three subcategories of the behavioral interaction domain: interactions with the environment, interactions with other animals, and interactions with humans (20).

### 4.2 Illness and injury within the Five Domains

Through the Five Domains Model lens, impaired welfare can stem from illness or injury associated with Domain 3 (Health) and subsequent impacts on Domain 5 (Mental State) through feelings of pain, malaise, weakness, breathlessness, nausea, and physical exhaustion. Furthermore, reduced feeding and drinking behaviors may occur from inappetence, reduced foraging motivation, or reluctance to compete at the feed bunk, which can lead to reduced feed and water intake (Nutrition Domain) and subsequent hunger and thirst (Mental State Domain). Similarly, ill and injured animals that develop a fever often display heat- or cold-seeking behaviors (Behavior Domain) which may be exacerbated by thermal extremes in the environment (Environment Domain), which can impact thermal comfort (Mental State Domain). Hence, the confluence of sickness behavior with the design and management of hospital and chronic pens has tremendous potential to impact cattle welfare and recovery positively or negatively.

In addition to the presence of illness or injury leading to impaired welfare, one must consider that the severity and length of the health impairment can also impact welfare (92). When grading the degree of welfare compromise in animals with “untoward organ-specific clinical signs with various effects,” Mellor notes that animals with no clinical signs have no welfare compromise, animals with minor/short-lived clinical signs have “low” welfare compromise, animals with marked/short-lived or moderate/longer lived clinical signs have “marked to severe” welfare compromise, and animals with extreme clinical signs, followed by death while conscious have the most severe level of welfare compromise (92). This variation in welfare compromise from none to severe based on the length (short vs. long) and severity (minor vs. marked) of clinical signs of disease can be applied to acute vs. chronic feedlot cattle. Acute cattle tend to have a shorter duration of health impairment, but that short duration may be filled with more marked/severe clinical signs. In comparison, chronic cattle tend to have a longer duration of health impairment, where clinical signs may be less severe. Of course, these trends may vary on a case-by-case basis. For example, an individual may initially experience an acute phase of short, intense health impairment followed by a failure to recover and a subsequent chronic phase of more prolonged, less intense impairment. Nevertheless, this concept of the level of illness or injury impacting the level of welfare compromise is helpful when evaluating the welfare of acute and chronic feedlot cattle.

### 4.3 Case study: applying the Five Domains Model

During a routine home pen check of healthy animals, a producer observed a steer presenting with open mouth breathing and coughing, a depressed attitude, and appetite loss (anorexia). The steer was flagged for further evaluation and walked to the treatment facility. After a temperature check and lung auscultation, the steer was diagnosed with acute BRDC and treated according to feedlot standard operating procedures (SOPs). Using Table 2, the producer then evaluated the steer’s current welfare status to decide if the steer should be returned to his home pen or moved to a hospital pen. Firstly, it was noted that the steer was well-conditioned with a body condition score (BCS) of 6 (good) on a 9-point scale (96). During recent pen checks, the steer was observed at the feed bunk and waterer, and the rumen appears to have feed present (good gut fill). This indicates that despite the potential decrease in feed and water consumption common during sickness (56, 74, 81), the steer seemed to be consuming feed and water. Thus, the evidence indicated that the Nutrition Domain was relatively unaffected despite the BRDC diagnosis. At the time of evaluation, high ambient temperatures represented thermal extremes and an impact on the Physical Environment Domain. This interacted with the Health Domain, as cattle with BRDC may have impaired pulmonary capacity (97). Since cattle in high environmental temperatures thermoregulate via evaporative cooling (95), this may inhibit their ability to cool their body (98). The open-mouth breathing observed during the pen check (which was not observed in other steers in the pen) suggested that Physical Environment and Health Domains were impacted, and the associated mental effect of thermal discomfort, overheating, and breathlessness, which is considered a significant animal welfare issue (99), were impacting the steer’s welfare. Other physical impairments stemming from the acute BRDC (Health Domain) were likely experienced through the Mental State Domain with negative mental affects such as lethargy and dullness, pain, and nausea. Finally, the steer was housed in a familiar home pen environment with a familiar social group and did not seem to be isolating itself from the group. This provided opportunities for positive mental affects such as affectionate sociability from familiar pen mates, and comfort and safety within a familiar environment. There was also no evidence of increased aggressive or agonistic behaviors in the pen or directed towards the ill steer, indicating that the increase in bullying or competition sometimes seen in ill animals was not occurring. Additionally, the physical environment of the home pen included well-maintained dirt mounds that provided a dry resting place and windbreak, which allowed the steer some level of agency in his ability to choose where in the pen he could best convalesce. Thus, there was no indication of negative mental affects in the Behavior Domain. However, the steer’s agency could be further advanced with additional food resources and a shade structure that would provide additional choices for needs during convalescence. Overall, the primary sources of negative mental affects stemmed from the Health Domain and Physical Environment Domain, and there were sources of positive mental affects stemming from the Behavior and Nutrition Domains. Since the negative mental affects were related to the BRDC-related clinical signs, and the positive mental affects from the location of the steer in his home pen, the producer decided it was in the best interest of the steer’s welfare to keep him in his home pen instead of moving him to a hospital pen. Thus, the steer was returned to his home pen after treatment.

**Table 2:** Application of the 2020 Five Domains Model (20) to evaluate the welfare of ill or injured feedlot cattle^1^.

Domain	Condition(s)^2^	Outcomes indicating positive welfare	Associated positive mental affects (Mental State Domain)	Outcomes indicating negative welfare	Associated negative mental affects (Mental State Domain)
Nutritional – “The water and food available to animals”	Water intake: (−) restricted (+) correct quantities	Presence at waterer; signs of good hydration status	Pleasures of drinking (quenching)	Dehydration [e.g. sunken eyes, dry mucous membranes (93)], absence from waterer; competition at waterer	Thirst; weakness from dehydration
	Feed intake: (−) restricted (+) correct quantities	Good body condition score; good gut fill (full rumen); presence at the bunk during feeding events	Satiety	Poor body condition score; no gut fill (empty rumen); competition at the bunk; absence from bunk during feeding events	Hunger; weakness from starvation
	Food variety & quality: (−) poor quality, low variety (+) high quality, high variety	Good body condition score; use of alternative sources of food (e.g., hay)	Pleasures of food tastes/smells/textures; masticatory pleasures	Poor body condition score; absence or lack of use of alternative food sources	Hunger; malaise from malnutrition; eating-related boredom
Physical Environment– “The impacts of physical and atmospheric conditions to which animals are exposed directly”	Pen flooring: (−) uncomfortable, unclean (+) comfortable, well maintained	Good mud score (18); ease of postural changes	Physical comfort, thermal comfort	Poor mud score (18); physical evidence of skin irritation; pain behaviors when moving or lying	Physical discomfort: musculoskeletal pain, skin irritation, difficulty of movement
	Thermal environment (−) thermal extremes (+) effective shelter and shade	Signs of thermal comfort, use of available shelter and shade resources	Thermal comfort	Signs of overheating [open mouth breathing, high respiration rate; (94)] or chilling [shivering, huddling; (95)]	Thermal discomfort: chilling, dampness, overheating
Health – “The impacts of injury, disease and different levels of physical fitness”	Injury (acute, chronic, husbandry mutilations): (−) present (+) absent	Absence of physical signs in injury	Comfort of good health and functional capacity	Physical signs of injury (presence of cuts or lacerations, lameness)	Pain (many types), breathlessness, debility, weakness, sickness, malaise, nausea, dizziness
	Illness (acute, chronic): (−) present (+) absent	Absence of clinical signs of disease		Clinical signs of disease (temperature, nasal discharge, depressed temperament, etc.)	
	Functional impairment (e.g. amputation, genetic, lung, heart, kidney, neural): (−) present (+) absent	Absence of functional impairment		Presence of functional impairment (may be the result of a previously resolved illness or injury)	
Behavioral Interactions – “Interactions with humans, the environment, and other animals”	Agency and interaction with the environment:				
	Environment-focused activity: (−) present (+) absent	Behavior (e.g. normal activity, utilizing/exploring pen space)	Interest, pleasant occupation; calm, in control; engaged by activity, focused	Behavior (e.g. low activity, not utilizing/exploring pen space)	Various combinations: startled by unexpected events, neophobia, hypervigilance, anger, frustration, negative cognitive bias
	Foraging opportunities (−) present (+) absent	Behavior (e.g. bunk use, exploration of the pen, use of alternative food sources)		Behavior (e.g. bunk use, exploration of the pen, lack of or disuse of alternative food sources)	
	Agency and interaction with other animals:				
	Significant threats and limits on threat avoidance, escape, or defensive activity: (−) present (+) absent	Behavior (e.g. low levels of agonistic or aggressive behaviors; opportunities for escape and use of refuges; no limitations on sleep/rest)	Secure, protected, confident	Behavior (e.g. presence of agonistic or aggressive behaviors); physical signs of targeted bullying (e.g., buller animals)	Anger, anxiety, fear, panic, insecurity, neophobia
	Animal-to-animal interactive activity (−) present, positive (+) absent, negative	Behavior (e.g., allogrooming, proximity to known conspecifics, other positive affiliative behaviors)	Affectionate sociability	Behavior (e.g., isolation, decreased positive social interactions and play)	Loneliness, depression, yearning for company; thwarted desire to play
	Agency and interaction with humans:				
	Animal handling (−) poor (+) good, utilizes low-stress handling methods	Human behavior (e.g., patient, gentle, quiet, confident, kind, empathetic, subtle pressure cues); cattle behavior (e.g., short flight distance, calm alertness, compliantly responsive, seeks contact).	Calm, confident, at east, feels in control; enjoys variety	Human behavior (e.g., impatient, shouting, uncertain, fearful, indifferent, harsh pressure cues); cattle behavior (e.g., long flight distance, hypervigilant, attack/fight, escape, avoidance, freezing, non-compliant)	Anxiety, fear, panic terror, neophobia; insecurity, confusion, uncertainty, persistent, unease; helplessness; pain from injuries; negative cognitive bias
	Caretaker aptitude: (−) inexperienced, untrained, unskilled (+) trained, experienced, skilled				

^1^The conditions and associated mental affects presented in this table do not represent a comprehensive list of all positive and negative welfare indicators, and no single outcome is a conclusive indicator of welfare state. This table should be applied on an individual animal, case-by-case basis, with careful consideration of how the listed (and un-listed) outcomes combine in a multi-modal approach to welfare assessment.

2 Negative conditions are preceded by (−); positive conditions are preceded by (+).

After a period indicated by feedlot SOPs, the steer was evaluated a second time to determine treatment success. Visual examination of the steer showed that clinical signs of BRDC were not improved. Additionally, the steer had a BCS of 5 (moderate), indicating it has lost some weight since treatment. Using Table 2, the producer noted that the main area of change from the previous evaluation was in the Nutrition Domain, with the decreased BCS indicating the steer may have been experiencing intermittent hunger (a negative mental affect). As this was the only noticeable change from the previous evaluation, and the steer still seemed to be able to move freely within his home pen’s physical and social environment without difficulty, the producer decided to give a second treatment and return the steer to his home pen.

At the third evaluation, it was clear that the steer was still not responding to treatment and had experienced a significant decline since the last treatment. The steer now had a BCS of 3 (thin), a visibly concave rumen (no gut fill), and a visibly sunken and dry orbital area, indicating he had not been eating or drinking enough to maintain body weight and hydration. Thus, he was experiencing moderate to severe negative mental affects (Mental State Domain) via hunger, dehydration, and potentially weakness from starvation associated with the Nutrition Domain. The steer was observed open mouth breathing even during early morning pen checks before ambient temperatures were high. This indicated potentially significant impairment in pulmonary capacity (Health Domain). Combined with a lack of shade structures in the pen to protect from thermal extremes (Physical Environment Domain) and the inability of the steer to exercise his agency by seeking these shade structures as needed (Behavior Domain), the steer was likely experiencing significant negative mental affect due to breathlessness, overheating, and helplessness from the inability to seek shade or other methods of thermal regulation. These negative mental affects likely outweigh the potential positive affects the steer was experiencing from the familiarity of his home pen and social environment. This third evaluation of the steer’s welfare using Table 2 led the producer to conclude that the current resources provided to the steer in the home pen were insufficient for him to recover or maintain his welfare during illness and that action needed to be taken. Thus, the producer decided to treat a third time and move the steer to the chronic pen for closer monitoring, and where additional resources such as shade, long-stem hay, corn-stalk bedding, and additional floor space were available.

At the fourth evaluation after being moved to the chronic pen, the steer seemed to be potentially on the road to recovery. Since the chronic pen had fewer cattle and was checked twice as often as the home pens, the producer noted that the steer had been spending much of his time either eating from the long-stem hay feeder or resting under the shade structure in the pen. His body condition score had improved slightly (BCS 4, moderate), and there was evidence of gut fill, indicating he had recently eaten. Thus, the Nutrition Domain was improved, and he was likely experiencing the positive mental effect of satiety. The physical environment of the chronic pen, which included additional bedding and shade structures, represented an improvement in the Physical Environment Domain via effective shelter and shade, and the steer could experience improved thermal comfort from utilizing these resources. His ability to exercise agency and make choices (Behavioral Interaction Domain) was improved through the increase in resources available in the pen (shade, hay, bedding, etc.), which provided the steer with opportunities to experience positive mental affects such as confidence and feeling in control. One potential risk from the move to the chronic pen was the change in physical and social environment, which could have prompted negative mental affects such as neophobia, anxiety, loneliness, and insecurity from the unfamiliar pen and pen-mates. Fortunately, there was no evidence of this, as the steer was observed integrating into the chronic pen well with no evidence of isolation, bullying, or competition for resources. Thus, overall the steer has overall positive changes in his welfare state indicated by the Five Domains. At this point, the producer will continue monitoring the steer to ensure his recovery continues, so they can make further interventions down the road if needed. It is important to note that while the steer recovered after moving to the chronic pen in this case, there are cases where this will not occur, and the animal may continue to decline. In that case, the information in Table 2 can serve as a guide for timely euthanasia decision-making. Producers should consider the balance between positive and negative mental affects, the length and severity of suffering, and the likelihood of recovery, and how these impact the animals’ overall quality of life.

## 5 Discussion

The aim of this review was to provide a comprehensive overview of the acquired knowledge regarding ill and injured feedlot cattle welfare, focusing on existing knowledge gaps and implications for hospital and chronic pen management and welfare assurance. During the preliminary literature search, 110 papers with mention of ill or injured feedlot cattle welfare were identified. While this number of papers at first seems to indicate that this has been a well-studied subject, a closer look shows that many of these papers made only one or two mentions of cattle welfare. Similarly, many papers were conducted on a healthy population and collected one or two health outcomes (such as morbidity or BRDC incidence). Thus, while these papers were flagged based on the literature search terms, the study’s primary goal or population of interest was not ill or injured feedlot cattle or cattle welfare. This suggests an opportunity to purposefully integrate animal welfare outcomes into study design, particularly for studies that include ill and injured feedlot cattle as the population of interest. The second and third literature searches, which focused on managing ill and injured feedlot cattle, resulted in only 12 papers that mentioned hospital-type pens and two that mentioned chronic pens. Hence, published research with direct implications for managing this vulnerable population to maintain their welfare is scarce. The preponderance of studies relating to BRDC was unsurprising, as it remains the primary cause of morbidity and mortality in U.S. feedlots, and some cattle populations do not respond to treatment (100, 101). Other less common etiologies such as lameness, digestive issues (e.g., bloat, acidosis), and buller-steer syndrome are also important for ill and injured cattle welfare, and regional and housing-related differences across feedlots can impact the prevalence of different etiologies in their cattle. For example, the prevalence of digital dermatitis (a lameness-causing disease) in cattle herds varies across operations (102, 103) and housing conditions (102, 104). Lameness is the most common reason for an animal to be railed (31), which indicates that it is an important condition to consider when dealing with chronic animal populations. Lameness is associated with pain and discomfort (105), and bloat is also a painful condition (106), which has direct impacts on animal welfare, especially given that pain mitigation is not always consistently given to ill or injured cattle in feedlots (24).

There is a need for more focused research on specific subpopulations of ill and injured cattle, to provide a sound foundation of knowledge that can be referred to create benchmarks for audits and welfare assurance programs. Chronic cattle populations have received the least research attention, and they can vary greatly from feedlot to feedlot, both in total number, diagnosis, and final dispositions, all of which can have implications for chronic cattle welfare. One descriptive epidemiological study for calves entering a central Saskatchewan feedlot in the Fall of 1998 reported that 1.3% (158 calves) become chronic cattle (27). The 2011 NAHMS data indicates that the prevalence of chronic cattle treated for respiratory disease may be as high as 3.6% (24). Updated epidemiologic data on ill and injured cattle populations in commercial feedlots, especially chronic cattle, would help characterize these populations and potential risk factors for future research.

Blakebrough-Hall et al. investigated the effects of BRDC on economic outcomes and concluded that as the number of BRDC treatments increased from 0 to ≥3, feed costs and total value at slaughter decreased linearly. Additionally, cattle treated ≥3 times for BRDC grew 0.7 kg/d less and had carcasses 39.6 kg lighter than cattle never treated for BRDC (107). These findings have implications for the economic impacts of chronic cattle, as receiving ≥3 treatments is a common definition for chronically ill cattle. During an assessment of chronic pens at five Iowa beef feedlots, it was estimated that costs associated with treating cattle with chronic BRDC can range from 85 to 105 USD, and for chronically lame cattle mean treatment costs were around 63.48 USD. Additionally, there was an average daily maintenance cost of approximately 6.80 USD per head per day in the chronic pen, and chronic BRDC cattle with mortality outcomes had an average net profit of −946.50 USD (108, 109). Thus, there is evidence that certain management decisions, such as the amount of time cattle spend in the chronic pen and incur a daily maintenance cost, can impact the economic returns of an individual chronic animal. Economic data can also help with euthanasia decision-making, as managing cull animals in the feedlot is an essential part of a marketing strategy that optimizes feeder cattle health, welfare, and performance while minimizing death and economic losses (29). Thus, developing and implementing evidence-based guidelines for managing ill and injured feedlot cattle could help strengthen both cattle welfare and economic outcomes in chronic pens by helping feedlots manage their chronic pens in a way that balances these two important outcomes.

There is also a need for a clearer understanding of the behavioral responses of ill and injured feedlot cattle, how these behaviors vary with etiology and disease severity, and the implications of these behavioral variations for cattle management and welfare. Most sickness behavior studies of feedlot cattle focus on cattle with respiratory disease, many with the goal of early detection of morbid cattle using behavioral changes (110) and technological tools (86, 111). While early disease identification is vital for implementing effective BRDC therapeutics (112) and various lameness conditions (113), greater scrutiny of sickness motivation is needed to better understand the trajectory of convalescence and recovery, together with associated opportunities to improve pen designs. For example, does adding additional food resources to a chronic pen (such as long-stem hay) benefit all chronic cattle or only cattle with certain etiologies? Do cattle with chronic BRDC benefit from heat-mitigating resources such as shade, misters, or sprinklers, and which of these is the most beneficial and economically viable to implement? There is also evidence that sickness behavior expression differs for cattle with differing severity of parasitic infections (82–84). However, no research has been done on if this is true for other diseases and injuries. Since disease severity can vary greatly in other illnesses and injuries besides parasitism and for acute vs. chronic cattle, this may have implications for the identification, management, and welfare of these cattle. Finally, additional areas of research such as pen design (e.g., shade, wind breaks, space, flooring, nutrition, commingling) and diagnostics and health protocols (e.g., diagnostic tools, precision livestock technologies, animal record management) should be investigated and validated in field-based settings to help understand the short- and long-term effects on promoting ill and injured cattle convalescence, recovery, and welfare. As this additional research leads to the development evidence-based guidelines for ill and injured cattle management, collaboration with industry stakeholders and feedlot professionals will be vital to successfully implement and refine guidelines to ensure they are practical and effective in commercial feedlot settings.

To the authors’ knowledge, this review represents the first time that the Five Domains Model has been applied as a framework to evaluate the welfare of ill and injured feedlot cattle. It is important to recognize that every feedlot is different and may have different needs and possible solutions that work for their operation. Short case studies documenting what has (and has not) worked for individual feedlots to manage their ill and injured cattle populations would add valuable information to the knowledge base. This is especially true for managing illnesses and injuries that are less reported than lameness and BRDC—such as blind cattle, cattle with digestive issues, and cattle with neurological issues. In addition, there are opportunities for research on management factors that are involved in managing ill and injured cattle populations, such as producer training, economics, and records, which may reveal synergies between animal care and feedlot operation productivity. Finally, the Five Domains Model has been used before to help develop welfare assessment guidelines (114), and this approach could also be used to aid in the development and improvement of feedlot audits and welfare assurance schemes that can properly assess feedlots on their management of ill and injured cattle.

Dissemination of knowledge gained to current and future veterinarians, producers, and feedlot personnel is vital to ensure meaningful improvements in chronically ill or injured feedlot personnel management and welfare. Ensuring that information is provided in an accessible format is vital. Hands-on learning experiences have been shown to be the preferred method of instruction for cattlemen (115). In a 2014 survey, feedlot managers reported that most of their information on lameness prevention came from feedlot veterinarians, nutritionists, and training seminars (116). Feedlot nutritionists indicated that peer-reviewed journals were of great importance in their information-seeking behaviors (117). Ensuring that key subjects pertaining to ill and injured feedlot cattle management and welfare are a part of the veterinary curriculum is also important. In a 2021 survey of 10   U.S. veterinary schools, the authors concluded that veterinary schools should consider incorporating more advanced euthanasia training programs into curriculum (118). A 2021 survey of representatives from eight veterinary schools in Australia concluded that while most universities covered relevant materials using a variety of methods, at two schools that relied solely on clinical cases not all students will be exposed to making euthanasia decisions (119). Literature suggests that flipped classrooms (120), hybrid learning (121), and competency-based approaches (122, 123) are all promising teaching strategies that could enhance veterinary student learning. Finally, Terrell et al. found that around 11% of feedlot managers used internet-based sources for information (116); social media may prove a valuable resource for teaching and engagement in agriculture topics in the near future (124). Ultimately, as research and knowledge generation on the important topic of ill and injured cattle management and welfare continues to grow, it is vital that dissemination of knowledge to and collaboration with current and future professionals in the feedlot industry is emphasized to maximize the positive impacts on cattle welfare.

## 6 Conclusion

In this literature review articles on the management and welfare of ill and injured feedlot cattle were identified. Most articles relating to ill and injured feedlot cattle welfare were conducted on a healthy population with one or two measured health outcomes, indicating that there is a need for studies focusing on ill and injured feedlot cattle as the population of interest. The even greater sparsity of papers on managing ill and injured feedlot cattle in specialized hospital or chronic pens further suggests that there is a need for published research with direct implications for managing this vulnerable population to maintain their welfare. BRDC is by far the most prevalent diagnosis for acutely ill feedlot cattle, and a small percent of these cattle will become chronically ill. While other diagnoses, such as lameness, digestive issues, and pneumonia, are less prevalent, they also have important implications for cattle welfare. Research is needed to better understand these conditions and their welfare impacts. Cattle with varying diagnoses and severity of conditions will display similar behaviors during convalescence, and these behavioral responses can be used to design facilities that accommodate cattle convalescent behavioral needs. Additional research is needed to provide evidence-based best practices for hospital and chronic pen design and management. Proper application of the Five Domains Model to individual cases can help producers identify impaired cattles’ feelings and experiences and subsequent welfare outcomes to aid in management decision-making and pen design. Ultimately, by outlining the current knowledge of ill and injured feedlot cattle and utilizing this knowledge to assess cattle welfare, this review provided an essential step towards the ultimate goal of strengthening the care of ill and injured feedlot cattle.

## Author contributions

ES: Conceptualization, Investigation, Writing – original draft, Writing – review & editing. GD: Conceptualization, Funding acquisition, Project administration, Writing – review & editing. RD: Conceptualization, Funding acquisition, Writing – review & editing. AJ: Conceptualization, Writing – review & editing. DT: Conceptualization, Writing – review & editing. SM: Conceptualization, Funding acquisition, Project administration, Supervision, Writing – original draft, Writing – review & editing.

## Footnotes

^1^https://grsbeef.org/
